# Nonoperative Management of Multiple Penetrating Cardiac and Colon Wounds from a Shotgun: A Case Report and Literature Review

**DOI:** 10.1155/2018/7839465

**Published:** 2018-01-24

**Authors:** Paula M. Jaramillo, Jaime A. Montoya, David A. Mejia, Salin Pereira Warr

**Affiliations:** ^1^Pablo Tobón Uribe Hospital, Department of Surgery, University of Antioquia, Medellin, Colombia; ^2^Gastrointestinal Surgery and Digestive Endoscopy, National Cancer Institute, Militar Nueva Granada University, Bogotá, Colombia; ^3^Pablo Tobón Uribe Hospital, Medellin, Colombia

## Abstract

**Introduction:**

Surgery for cardiac trauma is considered fatal and for wounds of the colon by associated sepsis is normally considered; however, conservative management of many traumatic lesions of different injured organs has progressed over the years.

**Presentation of the Case:**

A 65-year-old male patient presented with multiple shotgun wounds on the left upper limb, thorax, and abdomen. On evaluation, he was hemodynamically stable with normal sinus rhythm and normal blood pressure, no dyspnea, or abdominal pain. Computed tomography (CT) scan of the chest shows hematoma around the aorta without injury to the blood vessel wall with an intramyocardial projectile without pericardial effusion. CT scan of the abdomen showed pellets in the transverse colon and descending colon endoluminal without extravasation of contrast medium or intra-abdominal fluid. The patient remains hemodynamically stable, and nonsurgical procedure was established.

**Discussion:**

Patients with asymptomatic intramyocardial projectiles can be safely managed without surgery. Nonsurgical management is only possible in asymptomatic patients with trauma of the colon through close surveillance and with very selective patients since standard management is surgery.

**Conclusion:**

Nonsurgical management of cardiac trauma, as well as colon penetrating trauma, can be performed in carefully selected patients with proper clinical follow-up, imaging, and laboratory studies.

## 1. Introduction

Due to armed conflict over the last decades of the last century, there have been great changes in clinical management, as well as in morbidity and mortality due to penetrating trauma of the colon and cardiac trauma. The surgical approach was the procedure to follow in cardiac trauma, initially considered fatal due to exsanguination or cardiac tamponade. For penetrating colon injuries due to associated sepsis, surgical management remains valid. However, conservative management of many traumatic injuries of different wounded organs has progressed over the years, leading to less aggressive surgeries and more benefits for patients.

We present a case of successful nonsurgical management of a patient with multiple penetrating cardiac and colon wounds from a shotgun.

## 2. Case Report

A 65-year-old male was admitted to Pablo Tobón Uribe Hospital in Medellin, Colombia, approximately 12 hours after receiving multiple penetrating wounds from a shotgun in the upper left limb, thorax, and abdomen. He reported a prior history of left anterior thoracotomy due to stab wounds and several abdominal surgeries due to gunshots. On evaluation, he was hemodynamically stable with normal sinus rhythm and normal blood pressure, no dyspnea, or abdominal pain. Focused assessment with sonography for trauma (FAST) did not show hemopericardium or intra-abdominal fluid, normal hemoglobin, and leukocytes.

**Figure 1:**
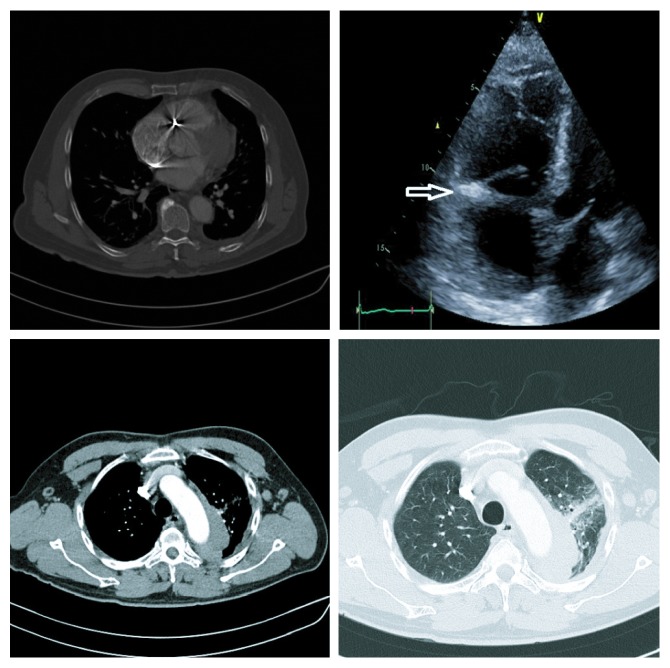
Thoracic CT shows hematoma around the aorta with no wall wound. Echocardiogram shows bullet in the tricuspid valve ring without perforation (arrow).

**Figure 2:**
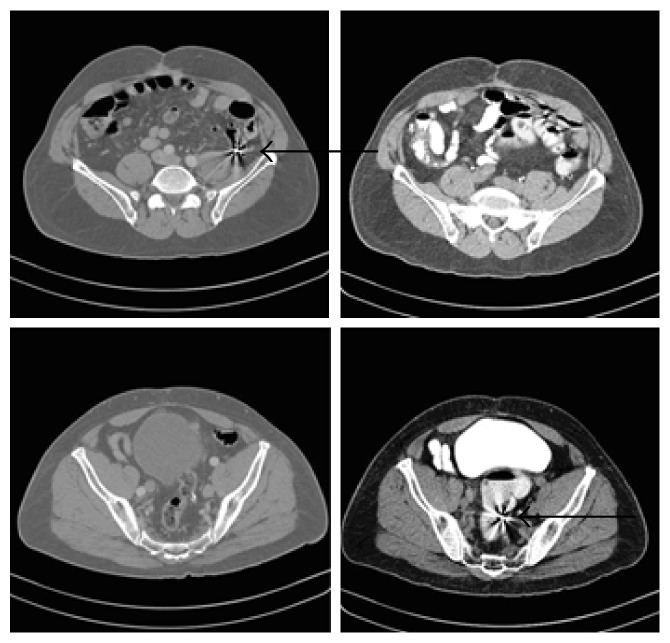
Abdominal computed tomography (CT) shows bullet fragments in the endoluminal descending colon (top arrow). Bullet migration located in the descending colon upon initial CT scan is now located in the rectal ampulla (bottom arrow).

Multidetector computed tomography (CT) of the chest and abdomen was performed to identify the trajectory of the pellets and to rule out associated wounds. A hematoma appeared around the aorta without injury to the blood vessel wall with an intramyocardial pellet without pericardial effusion. Echocardiogram showed fragments in the tricuspid valve ring without perforation (Figure 1). Abdominal CT showed three pellets: one located in the abdominal wall of the upper left quadrant, one in the area of the transverse colon, and the third in the area of the descending colon. It was not possible to determine whether the location was endoluminal, since no free liquid was observed in the abdomen (Figure 2). The patient remained hemodynamically stable, with no abdominal pain and no sign of cardiac tamponade. Nonsurgical treatment with critical care transfer, thoracic CT scan, and abdomen control were decided to determine changes in the mediastinum and heart. Additionally, oral and rectal contrast was performed to locate intra-abdominal bullets more accurately. Chest tomography scan shows hematoma around the aorta without changes in size and no pericardial effusion or pleural effusion (Figure 1). Abdominal CT scan showed fragments of the bullet in the transverse and descending endoluminal colon with no extravasation of the contrast medium or intra-abdominal fluid. In addition, bullet migration noticed in the descending colon during initial CT scan is now located in the rectal ampulla (Figure 2).

During the first 24 and 48 hours upon admittance to the hospital, the patient remains asymptomatic, and the oral procedure is started at 48 hours without abdominal pain or other symptoms. On the seventh day of hospitalization, he remains asymptomatic, CT scan of the thorax and abdomen shows no complications, and follow-up examinations like arterial gases and normal white cell count are taken as grounds to discharge the patient. Serial clinical examinations of the patient were performed by the same team, and we did not perform another echocardiography. The patient shows no associated complications in the follow-up carried out 30 days later and one year after the occurrence of the trauma.

## 3. Discussion

When a gunshot goes through tissue, it causes a temporary cavitation, which transmits energy producing laceration and rupture of the underlying tissue. There are high-velocity firearms in which the bullets travel at over 2000 ft/sec and transfer high energy to the tissues and low-velocity weapons in which the round travels below 1000 ft/sec, and the shotgun is an example of this type of weapon. The cartridge contains pellets that can vary in size from the very small (hundreds of pellets per cartridge) to those used in “combat shotgun” cartridges (nine or fewer large balls). Shotguns have an effective range of 30–50 metres. The injuries produced depend on whether the weapon is fired close to the target (pellets hit close together, creating one large wound) or at a distance (pellets have spread out, creating multiple small wounds) [1].

Low-speed fragments lose energy upon crossing the chest wall in the case of cardiac trauma and abdominal wall in the case of colonic trauma, causing reduction of the speed with which they impact the organ, with less trauma to the underlying tissue [2]. Both wounds presented by this patient were very critical, and the conventional treatment is surgical management.

Only 11% to 20% of patients with cardiac trauma arrive at the hospital with any vital signs, and, from these, the mortality rate is between 60% and 65% [3]. Clinical manifestations depend on the type of wound that may range from complete disruption of the wall, valvular injuries, and coronary vessels to partial lacerations of cardiac walls and myocardial contusions on the other side [4]. If the patient is hemodynamically unstable or with cardiac tamponade, like the patient who is stable, but hemopericardium is confirmed, the management is surgical with drainage of the pericardial sac followed by cardiorrhaphy. Nevertheless, possibility of nonoperative management with pericardial drainage has been considered in patients with hemodynamic stability, with no signs of cardiac tamponade, and with hemopericardium and absence of active bleeding [5].

Gunshot wounds of the heart are generally fatal and in most cases lead to exsanguination with few cases of cardiac tamponade (70–90% of these patients do not reach the ER). Wounds produced by multiple loads and low-speed weapons or fragmentation devices as in the case of military trauma (fragments of grenades, landmines, and bombs) produce injuries on a smaller scale in the pericardium or myocardium which may not require surgical management [6].

A pilot study in South Africa, conducted in 2001 and released in 2003, assessed 14 hemodynamically stable patients with evidence of hemopericardium. The study found that 71% of patients (10/14) had nontherapeutic sternotomy and that they could have received treatment only through pericardial drainage [7].

Subsequently, a prospective study also conducted in South Africa between 2001 and 2009 and released in 2014 collected information about 111 hemodynamically stable patients with hemopericardium without active bleeding; from these patients, 55 patients were randomly sent to sternotomy and 56 patients only to drainage. From the patients randomized to sternotomy, only four (7.3%) presented wounds compromising the entire cardiac wall, surpassing the endocardium which already had blood clots without evidence of bleeding. The remaining 92.7% had peripheral wounds or only pericardium affections. The conclusion of this study was that, in this type of selected patients, pericardial drainage is effective and safe compared to surgical management, since it reduces hospital and ICU stay, without increasing morbidity and mortality [8]. However, this type of management should only be performed in trauma centers with experience in managing heart wounds where surgical management is available when necessary.

Our patient was hemodynamically stable, asymptomatic, and without cardiac tamponade. He did not present pericardial effusion, probably due to adhesions of surgical procedures for previous trauma. The wound presented mediastinal hematoma without active bleeding or enlargement during observation, which allowed us to perform nonsurgical management.

Another factor to discuss was what to do with the pellet retained in the myocardium. The question was whether there was need for removal. In literature, we found case reports mostly related to military trauma. Almost all of these patients were asymptomatic, and finding bullets was incidental through imaging studies due to other causes [9].

Most literature reports on patients with retained intracardiac bullets are late in appearing after trauma and on completely asymptomatic patients. The need for removing bullets depends on clinical manifestations, location, and size thereof. Low-risk bullets are smaller than 5 mm and located completely in the myocardium, while those located in the pericardium are intracavitary, with a risk of infection from passing through contaminated areas or in patients with arrhythmia or valvular dysfunction, and should be removed [6, 10].

One of the most widely cited works in literature from the past 20 years is the one released in 1990 by Symbas and collaborators, who reviewed literature from 1940 to 1988 and their experience between 1968 and 1998, concluding that management should be treated individually, but that patients with asymptomatic intramyocardial bullets can be managed safely with no surgery [11]. The cardiothoracic surgeon's consultation is needed in complex cases of cardiac trauma.

Velmahos and Demetriades were the pioneers in selective nonoperative management of abdominal gunshot wounds, in which the serial physical examination, FAST, and CT scan play an important role defining the localization, type of injury, and if the patient requires surgical management. Patients with penetrating injuries to the left thoracoabdominal area are at high risk of diaphragmatic injuries. For patients without an indication for laparotomy, laparoscopy is considered a reasonable alternative to rule out diaphragmatic injuries, particularly if a larger than 2 cm laceration is suspected [12, 13].

Destructive colon injuries have produced dramatic improvements in both morbidity and mortality. Since the end of the last century, it was focused on less aggressive surgical management with suture and anastomosis, without the need of diversion [14].

In 1979, Stone and Fabian published a prospective study in which they compared primary closure versus colostomy, finding out that, in selected cases, the primary closure was better. However, they excluded patients with shock, blood loss greater than 20% of body mass, wounds in more than 2 intra-abdominal organs, significant contamination, and delay between diagnosis and surgery lasting more than 8 hours [15].

The current discussion remains upon what to do with patients with colostomy or primary closure, with multiple studies examining septic complications by comparing these two types of treatments [16–18]. In medical literature, there is only one case report of nonsurgical management of penetrating trauma of the colon caused by a low-speed bullet in a 19-year-old patient, with satisfactory hospital evolution and outpatient follow-up for 6 months [19].

In this patient, nonoperative management was performed since he arrived 12 hours after the trauma; he had no abdominal pain, and imaging studies showed no associated intra-abdominal complications. In addition, during clinical follow-up, evolution was satisfactory.

We consider that this type of management is only for highly selected patients, who are aware and have no traumas making clinical follow-up difficult (encephalocranial trauma or spinal cord injury).

## 4. Conclusion

Nonoperative management of cardiac trauma as well as colon penetrating trauma can be performed in asymptomatic patients, with close surveillance and selected patients since the standard management is surgery. It is a management option that the trauma surgeon should consider. However, there are not many reports supporting nonsurgical management in penetrating trauma of the colon, and surgical exploration remains the standard management.
